# 6,6′-Dimeth­oxy-2,2′-[(hexane-1,6-diyldi­oxy)bis­(nitrilo­methyl­idyne)]diphenol

**DOI:** 10.1107/S1600536808017789

**Published:** 2008-06-21

**Authors:** Wen-Kui Dong, Chun-Yu Zhao, Jin-Kui Zhong, Xiao-Lu Tang, Tian-Zhi Yu

**Affiliations:** a School of Chemical and Biological Engineering, Lanzhou Jiaotong University, Lanzhou 730070, People’s Republic of China; b Key Laboratory of Opto-Electronic Technology and Intelligent Control, Lanzhou Jiaotong University, Ministry of Education, Lanzhou 730070, People’s Republic of China

## Abstract

In the title compound, C_22_H_28_N_2_O_6_, strong intra­molecular O—H⋯N hydrogen bonds and weak inter­molecular C—H⋯O hydrogen bonds stabilize the three-dimensional supra­molecular structure.

## Related literature

For related literature, see: Akine *et al.* (2005 ▶); Costes *et al.* (2000 ▶); Dong *et al.* (2006 ▶, 2007 ▶); Duan *et al.* (2007 ▶); Hoshino (1998 ▶); Jacobsen *et al.* (1991 ▶); Katsuki (1995 ▶); Lacroix (2001 ▶); Srinivasan *et al.* (1986 ▶); Zhang *et al.* (1990 ▶).
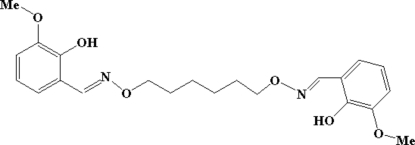


## Experimental

### 

#### Crystal data

C_22_H_28_N_2_O_6_
*M* *_r_* = 416.46
Monoclinic, 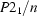

*a* = 6.2913 (9) Å
*b* = 29.063 (3) Å
*c* = 12.0481 (15) Å
β = 100.063 (2)°
*V* = 2169.0 (5) Å^3^
*Z* = 4
Mo *K*α radiation
μ = 0.09 mm^−1^
*T* = 298 (2) K
0.43 × 0.23 × 0.17 mm

#### Data collection

Bruker SMART CCD area-detector diffractometer
Absorption correction: multi-scan (*SADABS*; Sheldrick, 1996 ▶) *T*_min_ = 0.961, *T*_max_ = 0.984
10858 measured reflections
3836 independent reflections
2138 reflections with *I* > 2σ(*I*)
*R*_int_ = 0.042

#### Refinement

*R*[*F* ^2^ > 2σ(*F* ^2^)] = 0.047
*wR*(*F* ^2^) = 0.130
*S* = 1.08
3836 reflections
273 parameters
H-atom parameters constrained
Δρ_max_ = 0.17 e Å^−3^
Δρ_min_ = −0.14 e Å^−3^

### 

Data collection: *SMART* (Siemens, 1996 ▶); cell refinement: *SMART*; data reduction: *SAINT* (Siemens, 1996 ▶); program(s) used to solve structure: *SHELXS97* (Sheldrick, 2008 ▶); program(s) used to refine structure: *SHELXL97* (Sheldrick, 2008 ▶); molecular graphics: *SHELXTL* (Sheldrick, 2008 ▶); software used to prepare material for publication: *SHELXTL*.

## Supplementary Material

Crystal structure: contains datablocks global, I. DOI: 10.1107/S1600536808017789/hg2411sup1.cif

Structure factors: contains datablocks I. DOI: 10.1107/S1600536808017789/hg2411Isup2.hkl

Additional supplementary materials:  crystallographic information; 3D view; checkCIF report

## Figures and Tables

**Table 1:** Hydrogen-bond geometry (Å, °)

*D*—H⋯*A*	*D*—H	H⋯*A*	*D*⋯*A*	*D*—H⋯*A*
O5—H5⋯N2	0.82	1.95	2.662 (3)	145
O3—H3⋯N1	0.82	1.90	2.615 (3)	145

**Fig. 1.**
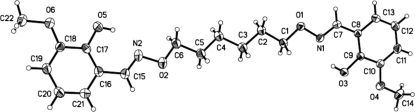
The molecule structure of (I) with atom numbering. Displacement ellipsoids for non-hydrogen atoms are drawn at the 30% probability level.

## supplementary crystallographic information

### Comment

Salen-type compound and its derivatives have attracted much attention to many organic as well as inorganic chemists, because these compounds can easily form complexes with various transition metal ions (Jacobsen *et al.*;1991, Katsuki *et al.*,1995). Some of them or their metal complexes are used as a catalyst in various organic reactions (Srinivasan *et al.*, 1986; Zhang *et al.*, 1990), nonlinear optical materials (Lacroix *et al.*, 2001), and metallomesogens (Hoshino *et al.*,1998) or exhibit interesting magnetic properties (Costes *et al.*, 2000) and so forth. To develop stable analogues of salen-type ligands, we synthesized a new class of salen-type bisoxime compounds on the basis of *O*-alkyl oxime moiety (–CH=N—O-(CH_2_)_n_-O—N=CH–) instead of the imine moiety (Dong *et al.*, 2006; Duan *et al.*, 2007). The larger electronegativity of oxygen atoms is expected to affect strongly the electronic properties of N_2_O_2_ coordination sphere, which can lead to different and novel properties and structures of the resulted complexes (Akine *et al.*,2005). Thus modification of a basic salen skeleton is very interesting and important. In this paper, a novel bisoxime ligand, 6,6'-dimethoxy-2,2'-[(hexane-1,6-diyldioxy)bis(nitrilomethylidyne)]diphenol (I) has been synthesized by 2 equiv. of 3-methoxysalicylidene and 1 equiv. of 1,6-bis(aminooxy)hexane, and shown in Fig. 1.

X-ray crystallographic analysis reveals the crystal structure of the bisoxime ligand (I), Which consists of discrete C_22_H_28_N_2_O_6_ molecules in which all bond lengths are in normal ranges. The dihedral angle of the two benzene rings is 20.9 (2)°. The oxime groups have anti-conformation, and there are strong O3—H3···N1 and O5—H5···N2 intramolecular hydrogen bonds and weak C7—H7···O3 and C22—H22A···C10 intermolecular hydrogen bonds, stabilize the three-dimensional supramolecuar structure of (I).

### Experimental

The title compound (I) was synthesized according to an analogous method reported earlier (Dong *et al.*, 2007). To an ethanol solution (5 ml) of 3-methoxysalicylidene (265.6 mg, 1.75 mmol) was added an ethanol (3 ml) solution of 1,6-bis(aminooxy)hexane (129.4 mg, 0.87 mmol). After the solution had been stirred at 328 K for 4 h, the mixture was filtered. The residue was washed successively with ethanol and ethanol/hexane (1:4), respectively. The product was dried under vacuum to yield 60.62 mg of (I). Yield, 16.7%. mp. 382–384 K. Anal. Calc. for C_22_H_28_N_2_O_6_: C, 63.45; H, 6.78; N, 6.73. Found: C, 63.47; H, 6.79; N, 6.61%.

Colorless prismatic single crystals suitable for X-ray diffraction studies were obtained after several weeks by slow evaporation from a mixture of ethanol/acetone (1:3) of (I) at room temperture.

### Refinement

Non-H atoms were refined anisotropically. H atoms were treated as riding atoms with distances C—H = 0.96 (CH_3_), or C—H = 0.97 (CH_2_), or 0.93 Å (CH), O—H = 0.82 Å, and *U*_iso_(H) = 1.2 *U*_eq_(C) and 1.5 *U*_eq_(O).

### Crystal data

**Table:**

C_22_H_28_N_2_O_6_	*F*_000_ = 888
*M**_r_* = 416.46	*D*_x_ = 1.275 Mg m^−^^3^
Monoclinic, *P*2_1_/*n*	Mo *K*α radiation λ = 0.71073 Å
Hall symbol: -P 2yn	Cell parameters from 2277 reflections
*a* = 6.2913 (9) Å	θ = 2.2–22.7º
*b* = 29.063 (3) Å	µ = 0.09 mm^−^^1^
*c* = 12.0481 (15) Å	*T* = 298 (2) K
β = 100.063 (2)º	Prismatic, colorless
*V* = 2169.0 (5) Å^3^	0.43 × 0.23 × 0.17 mm
*Z* = 4	

### Data collection

**Table:**

Bruker SMART CCD area-detector diffractometer	3836 independent reflections
Radiation source: fine-focus sealed tube	2138 reflections with *I* > 2σ(*I*)
Monochromator: graphite	*R*_int_ = 0.042
*T* = 298(2) K	θ_max_ = 25.0º
φ and ω scans	θ_min_ = 1.4º
Absorption correction: multi-scan(SADABS; Sheldrick, 1996)	*h* = −7→7
*T*_min_ = 0.961, *T*_max_ = 0.984	*k* = −34→34
10858 measured reflections	*l* = −10→14

### Refinement

**Table:**

Refinement on *F*^2^	Secondary atom site location: difference Fourier map
Least-squares matrix: full	Hydrogen site location: inferred from neighbouring sites
*R*[*F*^2^ > 2σ(*F*^2^)] = 0.047	H-atom parameters constrained
*wR*(*F*^2^) = 0.130	*w* = 1/[σ^2^(*F*_o_^2^) + (0.054*P*)^2^] where *P* = (*F*_o_^2^ + 2*F*_c_^2^)/3
*S* = 1.08	(Δ/σ)_max_ < 0.001
3836 reflections	Δρ_max_ = 0.17 e Å^−^^3^
273 parameters	Δρ_min_ = −0.14 e Å^−^^3^
Primary atom site location: structure-invariant direct methods	Extinction correction: none

### Special details

**Table:**

Geometry. All e.s.d.'s (except the e.s.d. in the dihedral angle between two l.s. planes) are estimated using the full covariance matrix. The cell e.s.d.'s are taken into account individually in the estimation of e.s.d.'s in distances, angles and torsion angles; correlations between e.s.d.'s in cell parameters are only used when they are defined by crystal symmetry. An approximate (isotropic) treatment of cell e.s.d.'s is used for estimating e.s.d.'s involving l.s. planes.
Refinement. Refinement of *F*^2^ against ALL reflections. The weighted *R*-factor *wR* and goodness of fit *S* are based on *F*^2^, conventional *R*-factors *R* are based on *F*, with *F* set to zero for negative *F*^2^. The threshold expression of *F*^2^ > σ(*F*^2^) is used only for calculating *R*-factors(gt) *etc*. and is not relevant to the choice of reflections for refinement. *R*-factors based on *F*^2^ are statistically about twice as large as those based on *F*, and *R*- factors based on ALL data will be even larger.

### Fractional atomic coordinates and isotropic or equivalent isotropic displacement parameters (Å^2^)

**Table:**

	*x*	*y*	*z*	*U*_iso_*/*U*_eq_	
N1	1.1005 (3)	0.21438 (7)	0.56103 (16)	0.0423 (5)	
N2	−0.1743 (4)	−0.00593 (7)	0.76432 (19)	0.0534 (6)	
O1	0.9073 (3)	0.19166 (6)	0.52040 (13)	0.0500 (5)	
O2	0.0109 (3)	0.02112 (6)	0.78379 (15)	0.0651 (6)	
O3	1.4536 (3)	0.23525 (5)	0.70115 (13)	0.0471 (5)	
H3	1.3377	0.2222	0.6810	0.071*	
O4	1.8128 (3)	0.27994 (6)	0.74024 (14)	0.0536 (5)	
O5	−0.5254 (3)	−0.04977 (6)	0.66091 (13)	0.0560 (5)	
H5	−0.4150	−0.0344	0.6644	0.084*	
O6	−0.8662 (3)	−0.09771 (6)	0.67802 (14)	0.0583 (5)	
C1	0.8437 (4)	0.16557 (8)	0.6096 (2)	0.0460 (7)	
H1A	0.8146	0.1861	0.6686	0.055*	
H1B	0.9593	0.1449	0.6416	0.055*	
C2	0.6460 (4)	0.13858 (9)	0.5642 (2)	0.0498 (7)	
H2A	0.6793	0.1166	0.5092	0.060*	
H2B	0.5355	0.1592	0.5263	0.060*	
C3	0.5602 (4)	0.11318 (9)	0.6573 (2)	0.0500 (7)	
H3A	0.6754	0.0944	0.6983	0.060*	
H3B	0.5203	0.1356	0.7096	0.060*	
C4	0.3675 (4)	0.08263 (9)	0.6171 (2)	0.0563 (7)	
H4A	0.2565	0.1004	0.5698	0.068*	
H4B	0.4104	0.0578	0.5718	0.068*	
C5	0.2762 (4)	0.06249 (9)	0.7145 (2)	0.0511 (7)	
H5A	0.3877	0.0444	0.7606	0.061*	
H5B	0.2386	0.0875	0.7607	0.061*	
C6	0.0802 (4)	0.03258 (9)	0.6804 (2)	0.0535 (7)	
H6A	−0.0319	0.0491	0.6306	0.064*	
H6B	0.1168	0.0051	0.6423	0.064*	
C7	1.1556 (4)	0.24225 (8)	0.4897 (2)	0.0425 (6)	
H7	1.0669	0.2460	0.4199	0.051*	
C8	1.3543 (4)	0.26824 (7)	0.51555 (19)	0.0375 (6)	
C9	1.4944 (4)	0.26352 (7)	0.61737 (19)	0.0364 (6)	
C10	1.6878 (4)	0.28817 (8)	0.6385 (2)	0.0399 (6)	
C11	1.7384 (4)	0.31800 (8)	0.5586 (2)	0.0508 (7)	
H11	1.8668	0.3346	0.5722	0.061*	
C12	1.5961 (5)	0.32325 (9)	0.4574 (2)	0.0577 (8)	
H12	1.6297	0.3437	0.4036	0.069*	
C13	1.4083 (5)	0.29895 (9)	0.4357 (2)	0.0520 (7)	
H13	1.3153	0.3028	0.3674	0.062*	
C14	2.0134 (4)	0.30314 (9)	0.7683 (2)	0.0622 (8)	
H14A	2.0954	0.2989	0.7090	0.093*	
H14B	2.0923	0.2908	0.8374	0.093*	
H14C	1.9881	0.3354	0.7777	0.093*	
C15	−0.2267 (4)	−0.01909 (8)	0.8565 (2)	0.0526 (7)	
H15	−0.1392	−0.0103	0.9235	0.063*	
C16	−0.4139 (4)	−0.04678 (8)	0.8628 (2)	0.0457 (7)	
C17	−0.5556 (4)	−0.06051 (8)	0.7667 (2)	0.0410 (6)	
C18	−0.7374 (4)	−0.08642 (8)	0.7769 (2)	0.0434 (6)	
C19	−0.7739 (4)	−0.09855 (9)	0.8825 (2)	0.0518 (7)	
H19	−0.8947	−0.1160	0.8894	0.062*	
C20	−0.6334 (5)	−0.08513 (9)	0.9779 (2)	0.0602 (8)	
H20	−0.6594	−0.0936	1.0488	0.072*	
C21	−0.4562 (5)	−0.05942 (9)	0.9683 (2)	0.0570 (8)	
H21	−0.3625	−0.0502	1.0329	0.068*	
C22	−1.0452 (4)	−0.12698 (9)	0.6835 (2)	0.0630 (8)	
H22A	−0.9947	−0.1555	0.7185	0.094*	
H22B	−1.1217	−0.1328	0.6086	0.094*	
H22C	−1.1402	−0.1123	0.7269	0.094*	

### Atomic displacement parameters (Å^2^)

**Table:**

	*U*^11^	*U*^22^	*U*^33^	*U*^12^	*U*^13^	*U*^23^
N1	0.0391 (13)	0.0454 (12)	0.0418 (12)	−0.0095 (10)	0.0050 (10)	−0.0060 (10)
N2	0.0475 (15)	0.0481 (13)	0.0663 (16)	−0.0098 (11)	0.0147 (12)	0.0014 (12)
O1	0.0465 (12)	0.0622 (11)	0.0398 (10)	−0.0191 (9)	0.0033 (8)	0.0022 (8)
O2	0.0556 (13)	0.0741 (13)	0.0667 (13)	−0.0256 (11)	0.0141 (10)	0.0043 (10)
O3	0.0448 (11)	0.0539 (10)	0.0411 (10)	−0.0083 (8)	0.0035 (8)	0.0085 (8)
O4	0.0394 (11)	0.0614 (11)	0.0544 (12)	−0.0089 (9)	−0.0073 (9)	0.0026 (9)
O5	0.0593 (13)	0.0662 (12)	0.0453 (11)	−0.0128 (10)	0.0167 (9)	0.0018 (9)
O6	0.0565 (13)	0.0676 (12)	0.0500 (12)	−0.0168 (10)	0.0070 (10)	0.0007 (9)
C1	0.0467 (17)	0.0468 (15)	0.0452 (16)	−0.0046 (13)	0.0100 (13)	0.0060 (13)
C2	0.0493 (17)	0.0531 (16)	0.0477 (16)	−0.0098 (14)	0.0100 (13)	0.0033 (13)
C3	0.0519 (18)	0.0507 (16)	0.0486 (16)	−0.0027 (14)	0.0124 (14)	0.0034 (13)
C4	0.0592 (19)	0.0590 (17)	0.0524 (17)	−0.0115 (15)	0.0144 (15)	0.0052 (14)
C5	0.0487 (18)	0.0516 (16)	0.0542 (17)	−0.0047 (14)	0.0122 (14)	0.0043 (13)
C6	0.0513 (18)	0.0515 (16)	0.0592 (18)	−0.0044 (14)	0.0136 (14)	0.0080 (14)
C7	0.0453 (17)	0.0452 (15)	0.0354 (14)	−0.0035 (13)	0.0023 (12)	−0.0006 (12)
C8	0.0431 (16)	0.0349 (13)	0.0346 (14)	−0.0037 (12)	0.0073 (12)	−0.0024 (11)
C9	0.0390 (16)	0.0345 (13)	0.0360 (14)	0.0005 (12)	0.0074 (12)	−0.0011 (11)
C10	0.0390 (16)	0.0387 (14)	0.0420 (15)	−0.0009 (12)	0.0067 (13)	−0.0046 (12)
C11	0.0482 (18)	0.0486 (16)	0.0565 (18)	−0.0137 (14)	0.0115 (15)	−0.0038 (14)
C12	0.071 (2)	0.0547 (17)	0.0498 (18)	−0.0166 (16)	0.0174 (16)	0.0079 (14)
C13	0.063 (2)	0.0549 (16)	0.0367 (15)	−0.0101 (15)	0.0058 (14)	0.0053 (13)
C14	0.0425 (18)	0.0679 (19)	0.072 (2)	−0.0089 (15)	−0.0016 (15)	−0.0131 (15)
C15	0.0488 (18)	0.0548 (17)	0.0531 (18)	−0.0098 (14)	0.0054 (14)	0.0035 (14)
C16	0.0481 (18)	0.0412 (15)	0.0489 (17)	−0.0027 (13)	0.0119 (14)	0.0012 (13)
C17	0.0466 (17)	0.0378 (14)	0.0417 (16)	0.0034 (13)	0.0164 (13)	0.0030 (12)
C18	0.0436 (17)	0.0427 (15)	0.0438 (16)	−0.0018 (13)	0.0073 (13)	−0.0020 (12)
C19	0.0516 (18)	0.0535 (16)	0.0526 (18)	−0.0097 (14)	0.0151 (15)	0.0024 (14)
C20	0.070 (2)	0.0666 (19)	0.0471 (18)	−0.0112 (17)	0.0197 (16)	0.0049 (15)
C21	0.064 (2)	0.0618 (18)	0.0434 (17)	−0.0077 (16)	0.0058 (15)	0.0012 (14)
C22	0.0527 (19)	0.0665 (18)	0.069 (2)	−0.0166 (16)	0.0082 (15)	−0.0045 (15)

### Geometric parameters (Å, °)

**Table:**

N1—C7	1.273 (3)	C6—H6B	0.9700
N1—O1	1.395 (2)	C7—C8	1.447 (3)
N2—C15	1.271 (3)	C7—H7	0.9300
N2—O2	1.391 (3)	C8—C9	1.387 (3)
O1—C1	1.428 (3)	C8—C13	1.397 (3)
O2—C6	1.429 (3)	C9—C10	1.397 (3)
O3—C9	1.360 (3)	C10—C11	1.373 (3)
O3—H3	0.8200	C11—C12	1.390 (3)
O4—C10	1.357 (3)	C11—H11	0.9300
O4—C14	1.418 (3)	C12—C13	1.362 (3)
O5—C17	1.357 (3)	C12—H12	0.9300
O5—H5	0.8200	C13—H13	0.9300
O6—C18	1.359 (3)	C14—H14A	0.9600
O6—C22	1.422 (3)	C14—H14B	0.9600
C1—C2	1.491 (3)	C14—H14C	0.9600
C1—H1A	0.9700	C15—C16	1.439 (3)
C1—H1B	0.9700	C15—H15	0.9300
C2—C3	1.518 (3)	C16—C17	1.391 (3)
C2—H2A	0.9700	C16—C21	1.392 (3)
C2—H2B	0.9700	C17—C18	1.393 (3)
C3—C4	1.512 (3)	C18—C19	1.377 (3)
C3—H3A	0.9700	C19—C20	1.379 (4)
C3—H3B	0.9700	C19—H19	0.9300
C4—C5	1.511 (3)	C20—C21	1.364 (3)
C4—H4A	0.9700	C20—H20	0.9300
C4—H4B	0.9700	C21—H21	0.9300
C5—C6	1.506 (3)	C22—H22A	0.9600
C5—H5A	0.9700	C22—H22B	0.9600
C5—H5B	0.9700	C22—H22C	0.9600
C6—H6A	0.9700		
			
C7—N1—O1	112.83 (19)	C13—C8—C7	119.3 (2)
C15—N2—O2	111.1 (2)	O3—C9—C8	122.9 (2)
N1—O1—C1	109.19 (17)	O3—C9—C10	116.5 (2)
N2—O2—C6	111.04 (19)	C8—C9—C10	120.6 (2)
C9—O3—H3	109.5	O4—C10—C11	125.1 (2)
C10—O4—C14	118.9 (2)	O4—C10—C9	115.0 (2)
C17—O5—H5	109.5	C11—C10—C9	119.8 (2)
C18—O6—C22	117.4 (2)	C10—C11—C12	119.5 (2)
O1—C1—C2	109.2 (2)	C10—C11—H11	120.3
O1—C1—H1A	109.8	C12—C11—H11	120.3
C2—C1—H1A	109.8	C13—C12—C11	121.0 (2)
O1—C1—H1B	109.8	C13—C12—H12	119.5
C2—C1—H1B	109.8	C11—C12—H12	119.5
H1A—C1—H1B	108.3	C12—C13—C8	120.4 (2)
C1—C2—C3	111.5 (2)	C12—C13—H13	119.8
C1—C2—H2A	109.3	C8—C13—H13	119.8
C3—C2—H2A	109.3	O4—C14—H14A	109.5
C1—C2—H2B	109.3	O4—C14—H14B	109.5
C3—C2—H2B	109.3	H14A—C14—H14B	109.5
H2A—C2—H2B	108.0	O4—C14—H14C	109.5
C4—C3—C2	114.7 (2)	H14A—C14—H14C	109.5
C4—C3—H3A	108.6	H14B—C14—H14C	109.5
C2—C3—H3A	108.6	N2—C15—C16	123.7 (3)
C4—C3—H3B	108.6	N2—C15—H15	118.2
C2—C3—H3B	108.6	C16—C15—H15	118.2
H3A—C3—H3B	107.6	C17—C16—C21	119.2 (2)
C5—C4—C3	111.8 (2)	C17—C16—C15	121.8 (2)
C5—C4—H4A	109.3	C21—C16—C15	119.0 (2)
C3—C4—H4A	109.3	O5—C17—C16	122.8 (2)
C5—C4—H4B	109.3	O5—C17—C18	117.4 (2)
C3—C4—H4B	109.3	C16—C17—C18	119.9 (2)
H4A—C4—H4B	107.9	O6—C18—C19	125.3 (2)
C6—C5—C4	114.6 (2)	O6—C18—C17	115.2 (2)
C6—C5—H5A	108.6	C19—C18—C17	119.5 (2)
C4—C5—H5A	108.6	C18—C19—C20	120.8 (3)
C6—C5—H5B	108.6	C18—C19—H19	119.6
C4—C5—H5B	108.6	C20—C19—H19	119.6
H5A—C5—H5B	107.6	C21—C20—C19	119.9 (3)
O2—C6—C5	104.9 (2)	C21—C20—H20	120.1
O2—C6—H6A	110.8	C19—C20—H20	120.1
C5—C6—H6A	110.8	C20—C21—C16	120.8 (3)
O2—C6—H6B	110.8	C20—C21—H21	119.6
C5—C6—H6B	110.8	C16—C21—H21	119.6
H6A—C6—H6B	108.8	O6—C22—H22A	109.5
N1—C7—C8	120.9 (2)	O6—C22—H22B	109.5
N1—C7—H7	119.5	H22A—C22—H22B	109.5
C8—C7—H7	119.5	O6—C22—H22C	109.5
C9—C8—C13	118.6 (2)	H22A—C22—H22C	109.5
C9—C8—C7	122.0 (2)	H22B—C22—H22C	109.5
			
C7—N1—O1—C1	−172.91 (19)	C10—C11—C12—C13	−0.6 (4)
C15—N2—O2—C6	175.0 (2)	C11—C12—C13—C8	0.3 (4)
N1—O1—C1—C2	−175.89 (18)	C9—C8—C13—C12	0.8 (4)
O1—C1—C2—C3	−175.30 (19)	C7—C8—C13—C12	−178.9 (2)
C1—C2—C3—C4	−176.4 (2)	O2—N2—C15—C16	178.7 (2)
C2—C3—C4—C5	−173.9 (2)	N2—C15—C16—C17	−1.4 (4)
C3—C4—C5—C6	178.6 (2)	N2—C15—C16—C21	179.8 (3)
N2—O2—C6—C5	178.64 (19)	C21—C16—C17—O5	−179.7 (2)
C4—C5—C6—O2	−174.7 (2)	C15—C16—C17—O5	1.5 (4)
O1—N1—C7—C8	−178.54 (19)	C21—C16—C17—C18	0.3 (4)
N1—C7—C8—C9	1.1 (4)	C15—C16—C17—C18	−178.5 (2)
N1—C7—C8—C13	−179.2 (2)	C22—O6—C18—C19	−4.5 (4)
C13—C8—C9—O3	178.6 (2)	C22—O6—C18—C17	175.5 (2)
C7—C8—C9—O3	−1.7 (4)	O5—C17—C18—O6	−0.6 (3)
C13—C8—C9—C10	−1.5 (3)	C16—C17—C18—O6	179.5 (2)
C7—C8—C9—C10	178.2 (2)	O5—C17—C18—C19	179.4 (2)
C14—O4—C10—C11	−0.6 (3)	C16—C17—C18—C19	−0.6 (4)
C14—O4—C10—C9	179.4 (2)	O6—C18—C19—C20	−179.7 (2)
O3—C9—C10—O4	1.1 (3)	C17—C18—C19—C20	0.4 (4)
C8—C9—C10—O4	−178.8 (2)	C18—C19—C20—C21	0.2 (4)
O3—C9—C10—C11	−178.9 (2)	C19—C20—C21—C16	−0.5 (4)
C8—C9—C10—C11	1.2 (3)	C17—C16—C21—C20	0.3 (4)
O4—C10—C11—C12	179.9 (2)	C15—C16—C21—C20	179.1 (2)
C9—C10—C11—C12	−0.1 (4)		

### Hydrogen-bond geometry (Å, °)

**Table:**

*D*—H···*A*	*D*—H	H···*A*	*D*···*A*	*D*—H···*A*
O5—H5···N2	0.82	1.95	2.662 (3)	145
O3—H3···N1	0.82	1.90	2.615 (3)	145
